# Pattern of p53 protein expression is predictive for survival in chemoradiotherapy-naive esophageal adenocarcinoma

**DOI:** 10.18632/oncotarget.22021

**Published:** 2017-10-24

**Authors:** Fiebo J.C. Ten Kate, Lucia Suzuki, Lambert C.J. Dorssers, Winand N.M. Dinjens, David T.W. Jones, Daan Nieboer, Michael Doukas, J. Jan B. Van Lanschot, Bas P.L. Wijnhoven, Leendert H.J. Looijenga, Katharina Biermann

**Affiliations:** ^1^ Department of Pathology, Erasmus University Medical Center, Rotterdam, The Netherlands; ^2^ Department of Public Health, Erasmus University Medical Center, Rotterdam, The Netherlands; ^3^ Department of Surgery, Erasmus University Medical Center, Rotterdam, The Netherlands; ^4^ Division of Pediatric Neurooncology, German Cancer Research Center (DFKZ), Heidelberg, Germany

**Keywords:** esophageal adenocarcinoma, p53, survival, mutational profile, DNA methylation

## Abstract

**Introduction:**

*TP53* mutations are considered to be the driving factor in the initiation of esophageal adenocarcinoma (EAC). However, the impact of this gene and its encoded protein as a prognostic marker has not been definitely established yet.

**Methods:**

In total, 204 chemoradiotherapy (CRT)-naive patients with EAC were included for p53 protein expression evaluation by immunohistochemistry (IHC) on the resection specimens, categorized as overexpression, heterogeneous or loss of expression, and correlated with disease free survival (DFS) and overall survival (OS) using multivariable Cox regression analysis. In a subset representing all three IHC subgroups mutational status of selected candidate genes (n=33) and high throughput methylation profiling (n=16) was assessed.

**Results:**

Compared to heterogeneous p53 expression, loss and overexpression were both independently predictive for adverse DFS and OS. *TP53* mutational status significantly correlated with the IHC categories (p=0.035). Most of the EAC with loss- or overexpression harbored *TP53* mutations (18/20, representing nonsense and missense mutations respectively). In contrast, 6/13 EAC with heterogeneous expression were *TP53* wild type, of which two demonstrated *MDM4* or *MDM2* amplification*.* Combined genomic hypomethylation and high frequency of intra-chromosomal breaks was found in a selection of EAC without p53 overexpression.

**Conclusion:**

P53 expression pattern is prognostic for DFS and OS in this historical cohort of CRT-naive EAC. P53 IHC is an informative readout for *TP53* mutational status in EAC with either loss- or overexpression, but not in case of a heterogeneous p53 pattern. Different EAC pathogenesis might exist, related to p53 and other candidate gene status, DNA hypomethylation and intrachromosomal breaks.

## INTRODUCTION

Esophageal adenocarcinoma (EAC), being rare before the second half of the 20^th^ century, is nowadays the predominant histological type of esophageal cancer in Western countries [1–3]. Presently the prognostication of patients with EAC is largely based on the TNM-classification supplemented with histological criteria [4]. Although this system has its value in the stratification of patients into prognostic groups [5], the outcome for an individual patient is still difficult to predict. This is demonstrated by the fact that up to 27% of the patients with stage IB develop disease recurrence while up to 24% of the patients with stage IIIA EAC will have no disease recurrence after intentionally curative surgery [5]. Therefore, prognostic biomarkers complementing the TNM classification are urgently needed.

The *TP53* gene (OMIM# 191170), first discovered more than 30 years ago, has a cell- and context dependent biological function. It has been reported that p53 is deregulated in most cancer types. Given its central role in the control of proliferation and senescence, it can be assumed to be the driving force of cancers of various types, including EAC [6–8]. Several types of stress can lead to p53 dysregulation. In EAC, mutations in *TP53* are detected early in the pathogenesis, likely linked to severe DNA damage in Barrett esophagus (BE) due to the reflux of mixed gastric and duodenal juice into the esophagus [9]. Recent genome wide studies proposed that EAC precursor lesions containing *TP53* mutations rapidly develop extensive chromosomal instability with subsequent oncogene activation [10–12].

Because of its dominant role in the development of EAC, p53 was also tested as a biomarker in EAC precursor lesions and in advanced EAC. There is growing evidence that p53 overexpression is related to dysplasia and independently predictive for progression in BE [13–18]. Overexpression is likely due to *TP53* mutations which stabilize the affected protein. “Absence” of p53 staining was described more recently in dysplastic BE [19]. This loss of expression is likely to be related to truncating mutations or to alternative, including epigenetic, mechanisms. Supporting the significance of the loss of expression, a recent IHC p53 study on a large prospective BE cohort revealed a significantly higher rate of progression to high grade dysplasia or EAC in low grade dysplasia harboring p53 overexpression and even higher in BE with absence of p53 expression [13].

In parallel to the EAC precursors, the results of the earlier investigations also suggested significance of p53 in relation to prognosis in advanced EAC [20–22]. However, strong conclusions cannot be drawn because of several limitations, including heterogeneity related to p53 IHC interpretation and patient selection. This may have influenced the outcome of these studies and as such the true biological effect of p53 in the context of disease progression may remain unidentified.

Therefore, the aim of this study was to examine the prognostic value of p53 in a well-defined group of chemo- and radio-therapy-naive EAC, using a validated IHC approach. To further investigate the putative mechanism(s) involved, a combinatory investigation of expression pattern, mutational status of *TP53* and a selection of other (relevant) genes, as well as high throughput profiling was performed in a subset of EAC.

## RESULTS

### Patient characteristics

Two hundred and sixteen (216) patients were initially identified to be eligible for this study. Of 12 patients, the formalin-fixed paraffin-embedded (FFPE) blocks could not be retrieved and were therefore excluded. From the remaining 204 patients with EAC the majority had a pT3-tumor (85.3%), tumor positive lymph nodes (79.4%) and negative resection margins (62.7%). Detailed patient and tumor characteristics are shown in Table 1 and Supplementary Table 1.

**Table 1:** Clinico-pathological characteristics for the 204 included patients with esophageal adenocarcinoma

	All patients n=204		p53 Loss (0%) n=54		p53 Heterogeneous (1-60%) n=36		p53 Overexpression (61-100%) n=114		p-value
	N	%	N	%	N	%	N	%	
Age at surgery									
Median	64.0		63.0		68.5		64.0		0.462
Range (IQR)	55.3-72.0		55.0-72.0		56.3-74.0		55.0-72.0		
Sex									
Male	174	85.3	51	25.0	29	14.2	95	46.6	0.337
Female	30	14.7	5	2.5	7	3.4	17	8.3	
Siewert classification									
Type 1	75	36.8	23	11.3	11	5.4	41	21.1	0.576
Type 2	129	63.2	33	16.2	25	12.3	71	34.8	
Pathologic T-stage									
pT2	27	13.2	9	4.4	3	1.5	16	7.8	0.556
pT3 or pT4	177	86.8	47	23.0	33	16.2	96	47.1	
Pathologic N-stage									
pN0	42	20.6	16	7.8	5	2.5	22	10.8	0.207
pN1 or more	162	79.4	40	19.6	31	15.2	90	44.1	
Histology grade									
Well	5	2.5	3	1.5	1	0.5	1	0.5	0.498
Moderate	80	39.2	22	10.8	15	7.4	43	21.1	
Poor	119	58.3	31	15.2	20	9.8	68	33.3	
Resection margin status									
pR0	128	62.7	33	16.2	22	10.8	73	35.8	0.714
pR1	76	37.3	23	11.3	14	6.9	39	19.1	
Alive after 60 months									
Yes	34	160.7	10	4.9	8	3.9	16	7.8	0.518
No	170	83.3	46	22.5	28	13.7	96	47.1	

### P53 expression correlates with overall - and disease free survival

**Figure 1:**
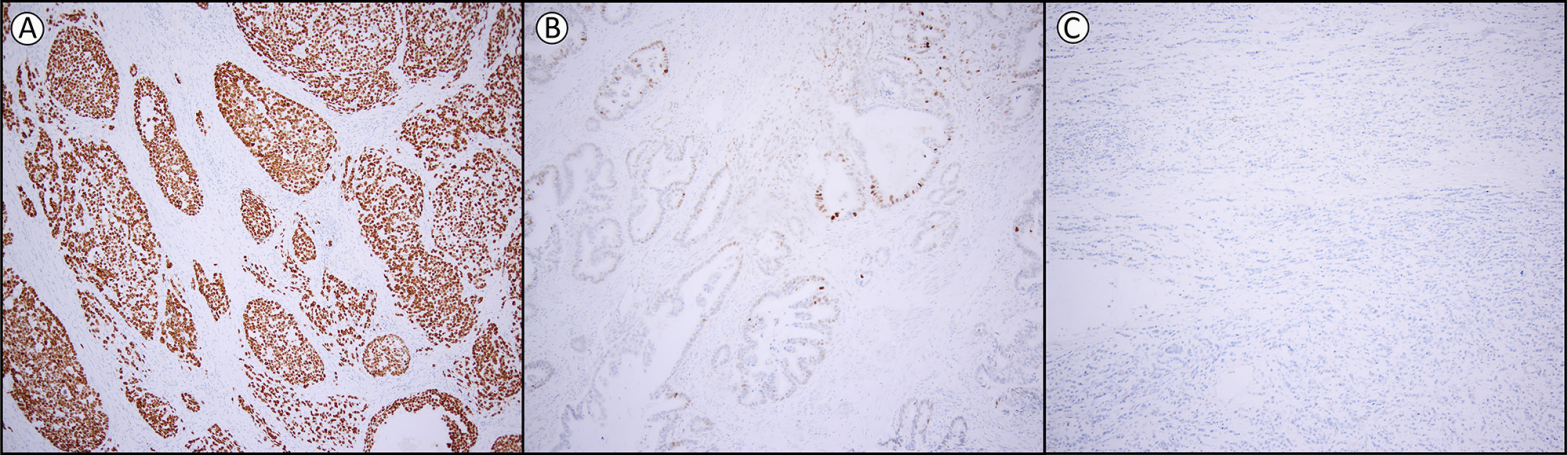
Examples of p53 expression in esophageal adenocarcinoma **(A)** overexpression (61-100% positive tumor cells) **(B)** heterogeneous expression (1-60% positive tumor cells) and **(C)** loss of expression (0% positive tumor cells). Magnification 1:100.

The optimal cut-off for p53 expression was calculated, based on the receiver operating characteristics (ROC) curve and Youden-index (see Supplementary Table 2 and Supplementary Figure 1), into three groups, namely loss of expression (0% of tumor cells positive), heterogeneous expression (1-60% of tumor cells positive) and overexpression (61-100% of tumor cells positive). The interobserver variation for the assessment of p53 between the two observers was excellent (kappa 0.850, p<0.001). From the 204 patients, 55.9% (n=114) of the EAC showed overexpression, 26.5% (n=54) loss of expression, while 17.6% (n=36) had a heterogeneous expression. In all cases this was a homogeneous expression pattern throughout the cancer, of which representative examples are shown in Figure 1.

**Figure 2:**
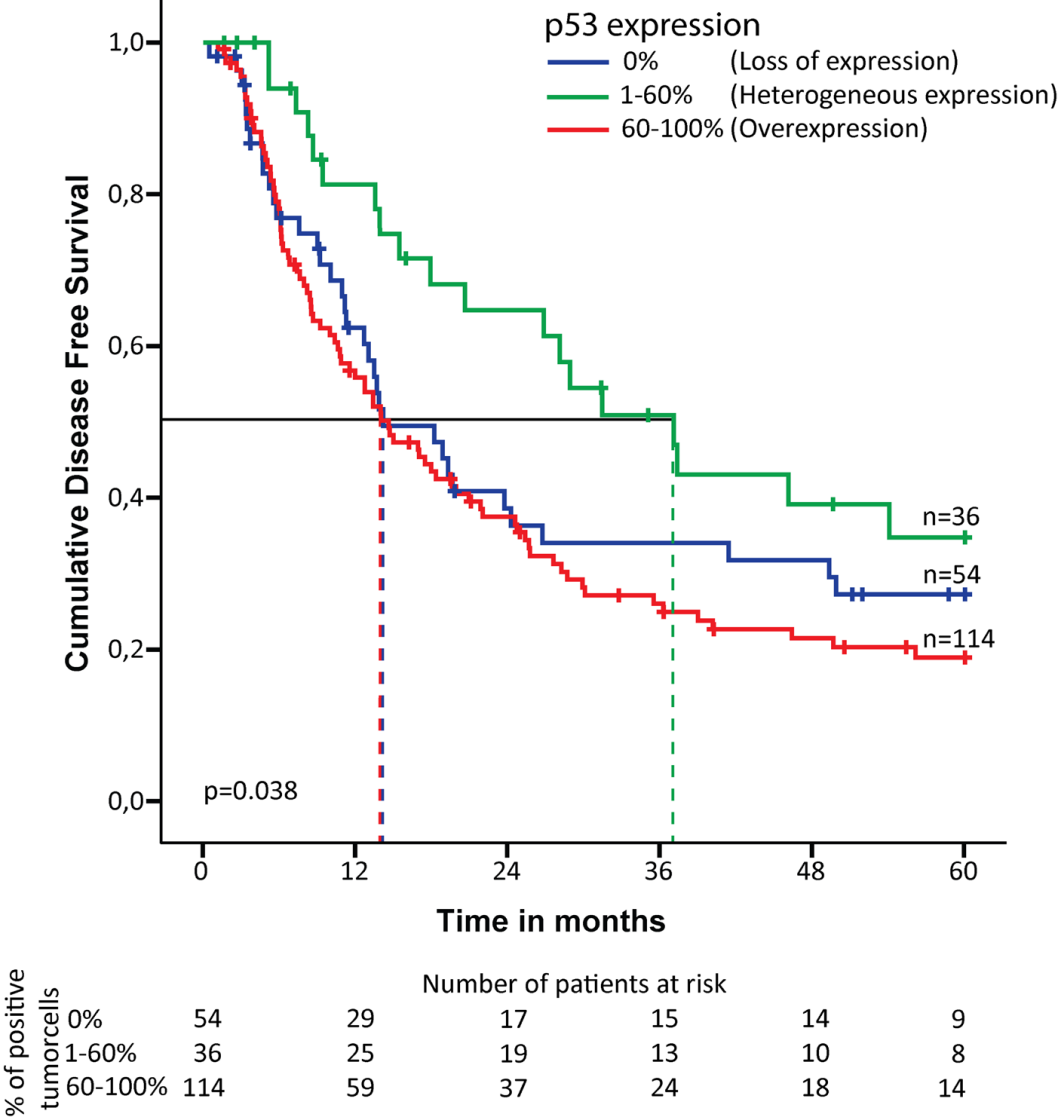
Kaplan-Meier curve for disease free survival in chemoradiotherapy-naive patients with esophageal adenocarcinoma Expression pattern of p53 is subdivided into three groups: 0% of the tumor cells positive (loss of expression), 1-60% of the tumor cells positive (heterogeneous expression) and 61-100% of the tumor cells positive (overexpression). The dotted line indicates the median survival for each of the three groups. Number of patients at risk is indicated for each of the three groups at the bottom of the figure.

The pattern of p53 expression associated with disease free survival (DFS); overexpression - median DFS 14.6 months (95% CI 10.0-19.2), loss of expression - median DFS 14.2 months (95% CI 7.9-20.5) compared to the group with heterogeneous p53 expression - median DFS 37.1 months (95% CI 24.3-49.9). The corresponding Kaplan-Meier curves are shown in Figure 2.

Univariable analysis demonstrated a correlation between p53 expression and DFS (p=0.036). The risk of recurrence of EAC was increased for patient with p53 overexpression (hazard ratio (HR) 1.91; 95% CI 1.16-3.14) as well as loss of p53 expression (HR 1.57; 95% CI 0.9-2.74) compared to heterogeneous p53 expression. This was also significant after multivariable analysis, adjusted for pT-stage, pN-stage, tumor differentiation and resection margin status (p=0.001). Patients with p53 overexpression/loss showed a significantly worse DFS compared to heterogeneous expression (HR 2.61; 95% CI 1.57-4.32; p= <0.001 and HR 2.75; 95% CI 1.55-4.9; p= <0.001, respectively) (Table 2 and Supplementary Table 3). A shorter overall survival (OS) was associated with p53 overexpression (median OS 19.4 months (95% CI 14.3-24.5)), and loss of expression (median OS 18.5 months (95% CI 15.3-21.7)) compared to the group with heterogeneous expression (median OS 32.4 months (95% CI 23.0-41.8)). Although no significance was identified in the univariable analysis (p=0.265), the multivariable analysis demonstrated that p53 expression was significantly associated with OS (p=0.003). Overexpression and loss of p53 expression were prognostic for a shorter survival period (HR respectively 1.99; 95% CI 1.29-3.07; p=0.002 and 2.17; 95% CI 1.33-3.55; p=0.002) compared to heterogeneous expression (Table 2 and Supplementary Table 3).

**Table 2:** Multivariable Cox regression analysis for disease free survival and overall survival in patients with esophageal adenocarcinoma

	Multivariable Cox regression analysis					
	Disease free survival			Overall survival		
	HR	95% CI	p-value	HR	95% CI	p-value
**Age**	NA	NA	NA	1.026	1.010-1.042	0.001
**pT-stage (ref pT2) pT3/4**	2.152	1.156-4.005	0.016	2.010	1.168-3.459	0.012
**pN-stage (ref pN0) pN+**	3.445	1.981-5.990	<0.001	2.434	1.560-3.796	<0.001
**Differentiation (ref good to moderate) poor**	1.467	1.016-2.119	0.041	1.551	1.112-2.165	0.010
**Resection margin (ref pR0) pR+**	1721	1.192-2.484	0.004	1.716	1.230-2.393	0.001
**p53 (ref heterogeneous) loss of expression overexpression**	2.754	1.547-4.903 1.571-4.320	0.001*	2.174	1.333-3.546	0.003*
	2.605			1.989	1.288-3.071	

HR=Hazard Ratio, CI=Confidence Interval, NA = not available, excluding patients who died within one month after surgery. P53 expression, based on immunohistochemical expression, was classified as loss of expression (0% of the tumor cells positive), heterogeneous expression (1-60% of the tumor cells positive) and overexpression (61-100% of the tumor cells positive). *global p-value

### Targeted mutational analyses and high throughput methylation profiling

**Figure 3:**
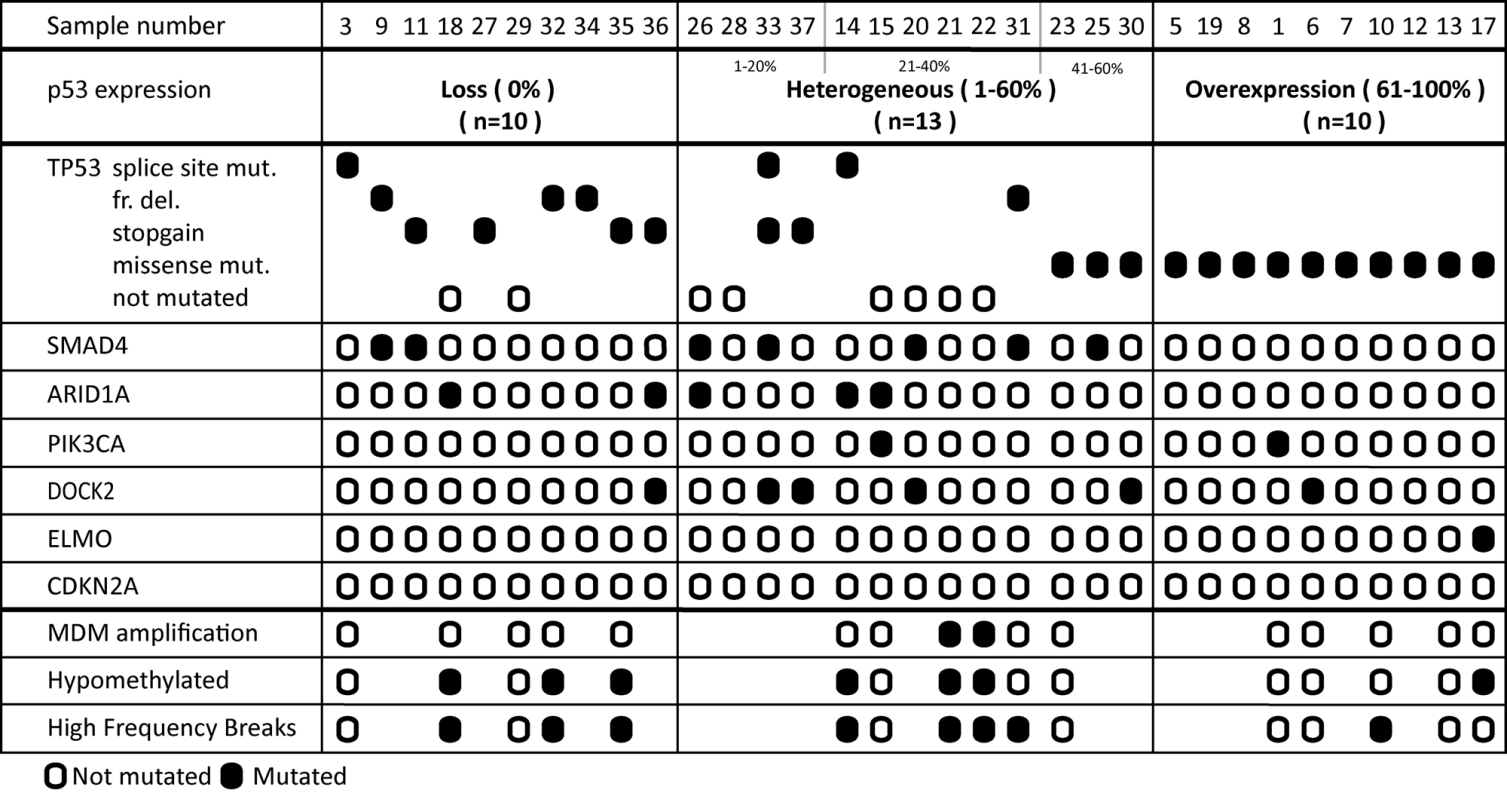
Molecular profile of esophageal adenocarcinoma Mutational profile, as detected by targeted sequencing in 33 cases is categorized by p53 expression pattern. The order of samples is determined by the percentage of positive p53 tumor cells. The exact mutations found are displayed in Supplementary Table 4, ordered by case number. The CpG methylation-derived information (copy numbers, hypo-methylation status and relative high frequency breaks are summarized here. Further details are provided in the Supplementary Figures 2 and 3.

**Figure 4:**
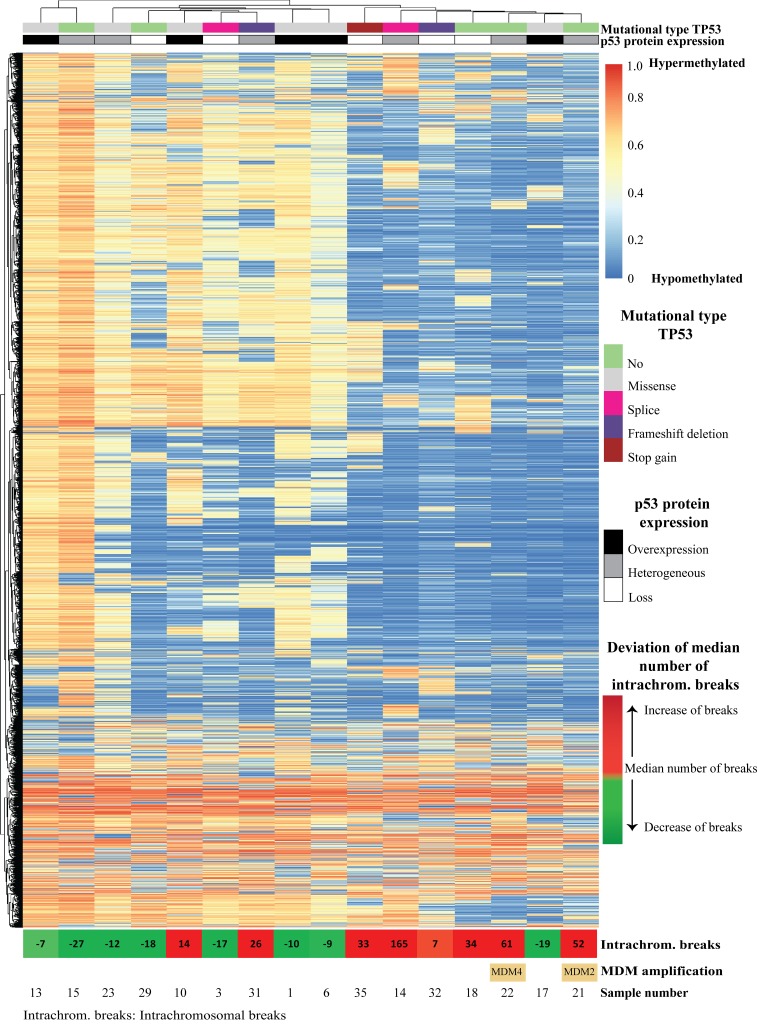
Unsupervised clustering of CpG methylation of 16 esophageal adenocarcinomas, using top 10,454 differential probes, in which every row is a methylation probe and every column is a sample The corresponding violin plots for all methylation probes as well as top 10,454 differential probes are depicted in Supplementary Figure 3. At the top of the image, p53 expression and *TP53* mutational status are indicated for each sample. In the bottom of the image, the deviation of the median number of intra-chromosomal breaks (median number of intra-chromosomal breaks are calculated, samples with less breaks are depicted in green, and samples with more breaks depicted in red, numbers indicate deviation from median number of intra-chromosomal breaks) as well as the two samples with *MDM2* and *MDM4*-amplification are indicated. Five samples with an altered p53 pathway (either *TP53* mutations or *MDM2/4* amplifications) show extensive intra-chromosomal breaks (sample 31, 35, 14, 22 and 21), and only one sample without alteration of the p53 pathway (sample 18) showed extensive intra-chromosomal breaks. However none of the six samples (sample number 13, 23, 10, 1, 6 and 17) with a missense mutation showed an increase of intra-chromosomal breaks. Increased number of intra-chromosomal breaks correlates to the clustered hypomethylated CpG-sites.

To shed light on the possible mechanism(s) underlying the p53 staining patterns, sequencing of the whole *TP53* gene was performed using the Ion Torrent platform on 33 selected EAC (10 with overexpression, 10 with loss, and 13 with a heterogeneous expression) (Supplementary Table 4). Overall, 25 of 33 (76%) EAC showed a *TP53* mutation. *TP53* status correlated significantly with the IHC staining pattern (p=0.035) (Figure 3 and Supplementary Table 5). Of the 10 cases with loss of expression eight had non-sense mutations (splice site, frameshift mutation or stopgain) and two no mutation. All EAC with overexpression of p53 as detected by IHC had missense mutations. The EAC within the heterogeneous p53 expression group demonstrated a mixed picture, representing the three different patterns. Those with more than 40% p53 positive tumor cells all showed missense mutations (n=3), in analogy to EAC with overexpression, while in the lower percentage category two out of four showed a nonsense mutation (one containing both a splice site and stopgain mutation). EAC cases with heterogeneous p53 expression in the middle group (n=6, 21-40%) demonstrated no underlying *TP53* mutations in four and two nonsense mutations. Besides *TP53*, in total, 21 other proven pathogenic mutations in the following genes *SMAD4* (n=7), *ARID1A* (n=5) , *PIK3CA* (n=2), *DOCK2* (n=6) and *ELMO* (n=1) were detected, significantly more in EAC with a heterogeneous p53 expression (13/21; p=0.032) (Figure 3 and Supplementary Table 5). In these samples no mutations in CDKN2A were detected. Four cases of our series revealed no mutation in the investigated genes (cases 21, 22, 28 and 29). Multiple mutations were identified (including *TP53*) in 15 EAC, predominantly again in the heterogeneous p53 expression group (9/13 versus 3/10 and 3/10, respectively). In addition, a subset of these EAC (n=16) were investigated using high throughput methylation profiling for the detection of chromosomal alterations between the three groups [11], including five with overexpression, five with loss and six with a heterogeneous expression, all with known *TP53* mutational status (see Figure 4). No hypermethylation of the promotor region of *TP53* was detected in any of these EAC, including the two cases with loss of p53 expression and wild type (not mutated) *TP53* (cases 18 and 29). Based on copy number variations (CNV) derived from these high throughput methylation profiles (see Material and Methods section), regional chromosomal amplifications were identified, including those encompassing for example *MDM2* and *MDM4*, two genes of which amplification is known to be related to an alternative inactivation of p53 besides mutations. Two EAC showed such an amplification (cases 21 and 22, for *MDM2*, confirmed by immunohistochemistry, and *MDM4*, respectively, See Supplementary Figure 2). No other mutations were identified in these cases, and both showed a heterogeneous p53 expression (21-40% of positive tumor cells) (Figures 3 and 4). Besides these specific amplifications, an unsupervised clustering of the top 10,454 most differentiating CpG-sites was performed (see Figure 4 (heatmap) and Supplementary Figure 3 (Violin plots)). No difference was identified for the overall methylation distribution between the EAC investigated (Supplementary Figure 3, bottom panel), while a clear hypomethylation profile was identified for the most differentiation CpG-sites in seven EAC out of the 16 cases. These included three with absence, three with a heterogeneous and one with overexpression of p53. Only one showed no *TP53* anomaly (case 18, no p53 expression), while all others demonstrated either a mutation in *TP53* itself (three nonsense, one missense), or amplification of *MDM2* or *MDM4*. In addition, the number of intrachromosomal breaks per individual EAC was scored based on the CNV profile (see Supplementary Figure 2 and Figure 4). This analysis demonstrated that six out of the seven EAC with a hypomethylation profile showed a higher number of breaks compared to the group median, i.e., indicated in red boxes in Figure 4 (including those with the *MDM2* and *MDM4* amplification), while this was observed for only two of the EAC within the non-hypomethylated group. These data suggest that there is a correlation between p53 status (protein expression, mutational profile and *MDM2/4* amplification), accumulation of other mutations (preferentially in the p53 heterogeneous staining group), preferential presence of a hypomethylated profile in the loss and heterogeneous p53 group, and occurrence of intrachromosomal breaks.

## DISCUSSION

This study primarily aimed to evaluate the relevance of p53 IHC for survival of patients with advanced EAC. A large, well defined cohort of CRT-naive surgically treated EAC was analyzed, and the pattern of p53 expression was shown to be significantly correlated with DFS and OS, independently from other clinico-pathological parameters including tumor stage. In addition, p53 expression patterns were correlated with the underlying *TP53* mutational status and genome wide methylation profile and derived information on chromosomal anomalies.

*TP53* is one of the driving genes for the progression of BE into adenocarcinoma and whole genome sequencing studies have detected a high mutation frequency of *TP53* in EAC [12, 23, 24]. Conflicting results have, however, been reported so far on *TP53* and survival in patients with EAC [21, 22, 25–30]. Three previous systematic reviews analyzed the current literature and performed a meta-analysis of up to 16 different studies, employing IHC or sequencing of the *TP53* gene [6–8]. Although, overall, similar results were reported in all three meta-analyses suggesting a negative effect of mutated *TP53* on prognosis, the data should be interpreted with caution. First of all, many of the earlier studies did not consider the bias of patient selection and chemoradiotherapy (CRT) treatment [20, 28, 31–33]. Several studies included patients who received surgery only as well as patients who underwent neoadjuvant treatment or definite CRT. This is of importance since p53 might modulate CRT response as suggested in earlier studies [20, 31–38]. Another important limitation of the published studies is the inconsistent methodology for detection and classification of p53 expression. From five studies using IHC on homogeneous EAC cohorts (total 384 patients), with surgery as single treatment modality and IHC approach, none qualified loss of expression as aberrant [21, 22, 26, 27, 29] (see Supplementary Table 6). This is significant since according to our interpretation, around 26% of EAC showed loss of p53 expression and had significantly worse outcome.

In the present study based on evaluation of EAC resection specimens of 204 CRT-naive patients, with surgery as single modality, p53 was detected by IHC and categorized by experienced observers using optimized cut-off values. The pattern was classified as heterogeneous, overexpression or loss of expression.

Until now it is not clear whether p53 IHC or sequencing of *TP53* is the most optimal tool to improve risk stratification in EAC. Mutational status was suggested to be preferable by a recent meta-analysis [7]. Several previous EAC studies applied mutational status as single read out [20, 22, 31]. The assays used for gene sequencing in those older studies are likely to be suboptimal, since the *TP53* gene was only partly sequenced using PCR-based methods, which correlates with the low mutational rate (40-50%) [20, 22, 31]. Although the efficacy of the gene sequencing techniques improved in recent years, they are still more time-consuming, labor intensive and expensive compared to IHC. Prediction of mutational status by IHC could be an alternative, but the prognostic accuracy might depend on the underlying cancer type [39]. To study the correlation between protein expression pattern and genetic status, a subset of 33 EAC was investigated using a targeted next generation sequencing approach. *TP53* mutational frequency rate was 76%, which is comparable to the recent investigations using whole genome or exome sequencing techniques [23, 24]. *TP53* status significantly correlated with the defined IHC categories (p=0.035). EAC with heterogeneous p53 expression was also heterogeneous in terms of the underlying *TP53* status, although it seems to be (again) subdivided into three groups, similar to loss of expression, similar to overexpression, and the (remaining) intermediate group. Of interest is that most additional mutations in the other candidate genes investigated were identified in the group with heterogeneous p53 expression, including two cases with regional amplifications of *MDM2* or *MDM4* (Figure 3). These were identified in EAC without any other mutation. In contrast, all EAC with high percentage of p53 positive cells (more than 61%, n=10) showed missense mutations in *TP53*, which is in line with results of two earlier studies [22, 40]. EAC with loss of p53 expression demonstrated predominantly nonsense mutations, including splicing, stopgain and frameshift mutations (8/10). These nonsense mutations were also observed in a subset of EAC with a heterogeneous, but relatively low to modest p53 expression, in fact three out of five cases. In 4 out of five of the remaining cases no *TP53* mutation was found. These observations warrant additional studies to be performed.

The putative difference in pathogenesis between these subgroups is supported by the results of the high throughput methylation profiling performed. The hypomethylated profile of the most differentiating CpG sites combined with a high frequency of intrachromosomal breaks was predominantly observed in EAC with loss or a heterogeneous p53 pattern (either by a nonsense mutation (n=3) or *MDM2/4* amplification (n=2)). No apparent differences were observed using all CpG targets, demonstrating its specificity. EAC with a hypomethylated profile showed a higher frequency of intrachromosomal breaks, indicative for chromosomal instability. This is in line with the recently suggested role of DNA methylation as the newly identified guardian of the genome [41]. Based on this small subset of patients, these observations might be a potential explanation for the differences in DFS and OS as found in the present study, which warrants further investigations. Besides the prognostic effect of p53 expression, our results are clinically important. *TP53* status might be predictive for response to neoadjuvant chemotherapy [20, 42]. Clinical trials, such as the PANCHO trial, stratified for *TP53* status, are underway and have completed recruitment [43]. Other studies rely on new therapeutic agents created to restore the wild type activity of p53, one of the most promising compounds being APR-246 [44]. Here we show that if IHC is used as a read-out for mutational status, results should be interpreted with caution especially in EAC with a heterogeneous p53 expression. In contrast, EAC with p53 overexpression or loss of expression are likely to have an underlying somatic mutation and extensive sequencing might not be necessary.

There are some limitations to this study. *TP53* sequencing was done in a single EAC area, and therefore potential intratumoral heterogeneity was not accounted for. However, this is considered unlikely to play an important role, since identical *TP53* mutations and homogeneous loss of heterozygosity of the *TP53* locus were detected across separated tumor regions in EAC previously [45], and a homogenous IHC was identified in all cases. Furthermore, although p53 is stained using a proven informative automatic staining system and a standardized protocol, the scoring is subjective in nature. However, the interobserver variation for p53 IHC was excellent.

In summary, this study leads to various conclusions. First of all, we have demonstrated that p53 expression pattern is significantly correlated with DFS and OS. This finding stresses the biological role of p53 for the prognosis of patients with EAC. Secondly, we have shown that IHC is a good read out for the presence of *TP53* mutations mainly in EAC with p53 overexpression and probably in EAC with loss of expression but not in EAC with a heterogeneous p53 expression. This might be important for current and future studies in which patient treatment is stratified according to the *TP53*/p53 status. In addition, our study could suggests existence of different pathogenesis of EAC, related to the p53 pathway (*TP53* mutational status and *MDM2*/*4* amplification), with downstream additional mutations of other candidate genes, as well as DNA methylation alterations and possibly related chromosomal instability. Yet, more work needs to be done for accurate genetic classification of EAC to fully reveal prognostic genetic signatures and involved mechanisms.

## MATERIALS AND METHODS

### Patient selection

To evaluate the prognostic value of p53 in patients with EAC, a cohort of patients who underwent surgery with curative intent between 1995 and 2006, without prior (neo-)adjuvant treatment, was selected from the Department of Surgery at the Erasmus University Medical Center (Rotterdam, The Netherlands). All patients had pathologically proven pT2-pT4a adenocarcinoma of the esophagus or at the gastro-esophageal junction. Only patients who were alive one month after surgery were included in the analysis to correct for surgical mortality. Clinical and pathological data were prospectively collected, including anatomical tumor location according to Siewert [46], tumor grade, pathological stage, age at surgery, comorbidities, OS and DFS. Tumor grading and staging was performed according to the TNM system as described by the UICC (Union Internationale Contre le Cancer, 2009, 7^th^ edition) [4]. Resection margin positivity was assessed on tumor cells in the resection margin. To ensure reliable classification, all slides were reviewed by an experienced GI pathologist (FK or KB) for depth of invasion.

The hematoxylin-eosin colored slides from the resection specimens were retrieved from the archive of the Department of Pathology at the Erasmus University Medical Center and a representative slide with EAC was selected. The corresponding FFPE block was retrieved and serial 4μm sections for IHC and mutational analysis were mounted on glass slides.

### Immunohistochemical analysis

The first slide of each selected FFPE block was stained for p53, ready to use kit (clone BP53-11, Ventana Medical Systems, Roche, Tuscon, AZ, USA). Staining was performed using an automated slide staining system (BenchMark Ultra, Ventana Medical Systems, Roche, Tuscon, AZ, USA), in which the slides were deparaffinized prior to the staining procedure and heat induced epitope retrieval at 97°C for 8 minutes. The primary antibody was incubated for 4 minutes, after which this was visualized using Ultraview (Ventana Medical Systems, Roche, Tuscon, AZ, USA) and counterstained with hematoxylin.

For optimal interpretation, representative tumor samples were evaluated by two experienced gastro-intestinal (GI) pathologists (KB and FK) with specific knowledge on p53, based on earlier published extensive IHC studies on EAC and its precursor lesions [13, 42]. A tumor sample with known overexpression of p53 was placed as positive control on each slide. Furthermore, normal tissue surrounding the tumor cells were evaluated for their physiological expression of p53, serving as internal control for the sample under investigation. If the positive control material or internal control was negative the slide was disregarded for analysis. The pattern of p53 IHC was scored on all tumors cells present on the slide, based on the percentage of tumor cells with nuclear positivity on a semi-quantitative 7-point scale: 0%, 1-20%, 21-40%, 41-60%, 61-80%, 81-90% and 90-100% of the tumor cells. If the scores of the two pathologists were discordant, a third board certified pathologist evaluated the slides (MD), after which the final diagnosis was based on the consensus of two of the three pathologists. All pathologists were blinded for clinical and pathological data.

### Mutational analysis and high throughput methylation profiling

In total 34 EAC, among them 10 with no expression of p53, 14 with heterogeneous expression (1-60% of the tumor cells positive) and 10 with overexpression (61-100% positive tumor cells), were selected for targeted gene sequencing. Tumor area was manually macro-dissected from the successive unstained slides, resulting in at least 30% tumor cells. DNA was extracted using proteinase K and 5% Chelex 100 resin [47]. An Ion AmpliSeq custom-made panel was created for selection of genes [45]. This consisted of primers for the entire *TP53* gene supplemented with hotspots or the entire genes known to be frequently altered in EAC (*ARID1A*, *PIK3CA*, *APC*, *DOCK2*, *ELMO1*, *CDKN2A* and *SMAD4*) [10–12, 23]. Sequencing was performed on the Ion Torrent Personal Genome Machine or IonS5 system (Thermofisher Scientific, Hemel Hempstead, UK) according to the manufacturers protocol. In short, libraries were created using the ION AmpliSeq Library Preparation Kit. Template was prepared using the Ion Onetouch Template Kit and sequencing was performed with the Ion Sequencing Kit as described [47]. One sample was excluded from further analysis because of poor DNA quality and high frequencies of formalin artefacts. All other samples showed comparable and reliable sequence read coverage independent from sample age. The sequence variants with a read frequency of less than 5% (homozygous reference) or more than 95% (homozygous non-reference), with an amplicon coverage of less than 50, or a variant coverage of less than 10 reads were excluded from analysis, to eliminate formalin artefacts. All variants found in an intronic, intergenic, non-coding RNA or UTR3/5 region, and synonymous single nucleotide variations (SNV) were excluded.

Sixteen EAC, among them five tumors with loss of expression, five with overexpression and six with heterogeneous p53 expression, were selected for genome-wide methylation analysis in addition to the targeted sequencing. Therefore, the Infinium MethylationEPIC BeadChip (Illumina, San Diego, CA, USA), targeting over 850,000 methylation sites, was applied according to the manufacturer’s instruction at the Microarray unit of the Genomics and Proteomics Core Facility of the German Cancer Research Center (DKFZ, Heidelberg, Germany). For a detailed description see earlier publication [48]. For unsupervised clustering the most differential probes (with 0,22 SD difference from the mean) were selected. To assess copy number variation (CNV) methylation data were implemented in the R/Bioconducter packages Conumee. Intra-chromosomal breaks were calculated from the number of segments defined by the Conumee package (blue horizontal lines in Supplementary Figure 3). Segments are defined as chromosomal regions with distinct copy number changes to the adjacent region. The number of segments relative to the median number of segments within this sample series was determined for each sample (presented in Figure 4). With this method amplification of genes were also assessed as described earlier.[49] To validate amplification of MDM2 immunohistochemistry staining (clone 1F2, Merck Milipore, Amsterdam, Holland) was performed on all samples in which no TP53 mutation was found.

### Ethics

The investigational protocol was approved by the medical ethical committee in the Erasmus Medical Center (Rotterdam, The Netherlands) (MEC-12-469).

### Statistical analysis

The primary endpoint of the study was 5-year DFS, defined as the time between surgery and the first clinical recurrence of disease, defined as clinical or radiological evidence of disease recurrence. Patients lost to follow-up were censored at the time of the last visit to the outpatient clinics. Secondary endpoint was OS, defined as time between surgery and death. The optimal cut-off for IHC was calculated using a ROC-curve and corresponding Youden-index (Supplementary Figure 1 and Supplementary Table 2).

The interobserver variation for the assessment of p53 staining between the two pathologists was calculated using Cohen’s kappa. Strength of agreement was categorized as follows: 0.00–0.20, poor; 0.21–0.40, fair; 0.41–0.60, moderate; 0.61–0.80, good; and 0.81–1.00, excellent.

Kaplan Meier curves were used to plot the 5-year DFS by p53 status. Uni- and multivariable Cox proportional hazard models were applied to calculate the association between p53 IHC and survival. In the multivariable analysis adjustments were made for all clinical and pathological factors which proved to be prognostic for survival in the univariable analysis (p<0,05). The pN-stage was dichotomized in pN0 and a pN+ (pN1-3) group for the Cox regression analysis. The p53 status and mutational status were correlated using Fisher’s Exact test. The analysis was performed using SPSS-software (version 22, SPSS IBM inc, Armonk, NY, USA). A p-value of <0.05 was considered statistically significant.

## SUPPLEMENTARY MATERIALS FIGURES AND TABLES





## Abbreviations

BE: Barrett esophagus
CRT: chemoradiotherapy
CNV: copy number variation
DFS: disease free survival
EAC: esophageal adenocarcinoma
FFPE: formalin-fixed paraffin-embedded
GI: gastro-intestinal
HR: hazard ratio
IHC: immunohistochemistry
LGD: low grade dysplasia
OS: overall survival
ROC: receiver operating characteristics
SNV: Single Nucleotide Variations
