# Supplementary material for: Pattern of p53 protein expression is predictive for survival in chemoradiotherapy-naive esophageal adenocarcinoma
Source: Oncotarget. 2017 Oct 24;8(61):104123–35. doi: 10.18632/oncotarget.22021 (PMC5732792; doi:10.18632/oncotarget.22021)
Supplement: Supplementary file 1 [file oncotarget-08-104123-s001.pdf]

Pattern of p53 protein expression is predictive for survival in chemoradiotherapy-naive esophageal adenocarcinoma

SUPPLEMENTARY MATERIALS

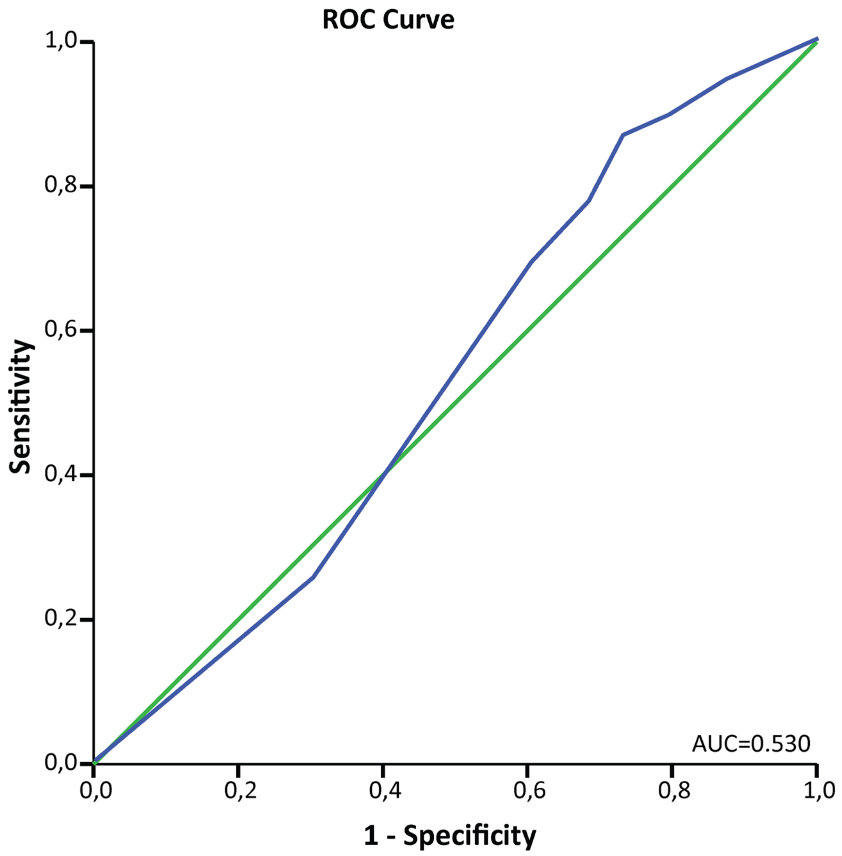

Supplementary Figure 1: Receiver operating characteristics-curve for the semi-quantitative p53 expression, according for the % of nuclear positive tumors cells, which is used to calculate the optimal cut-off value of p53 expression.

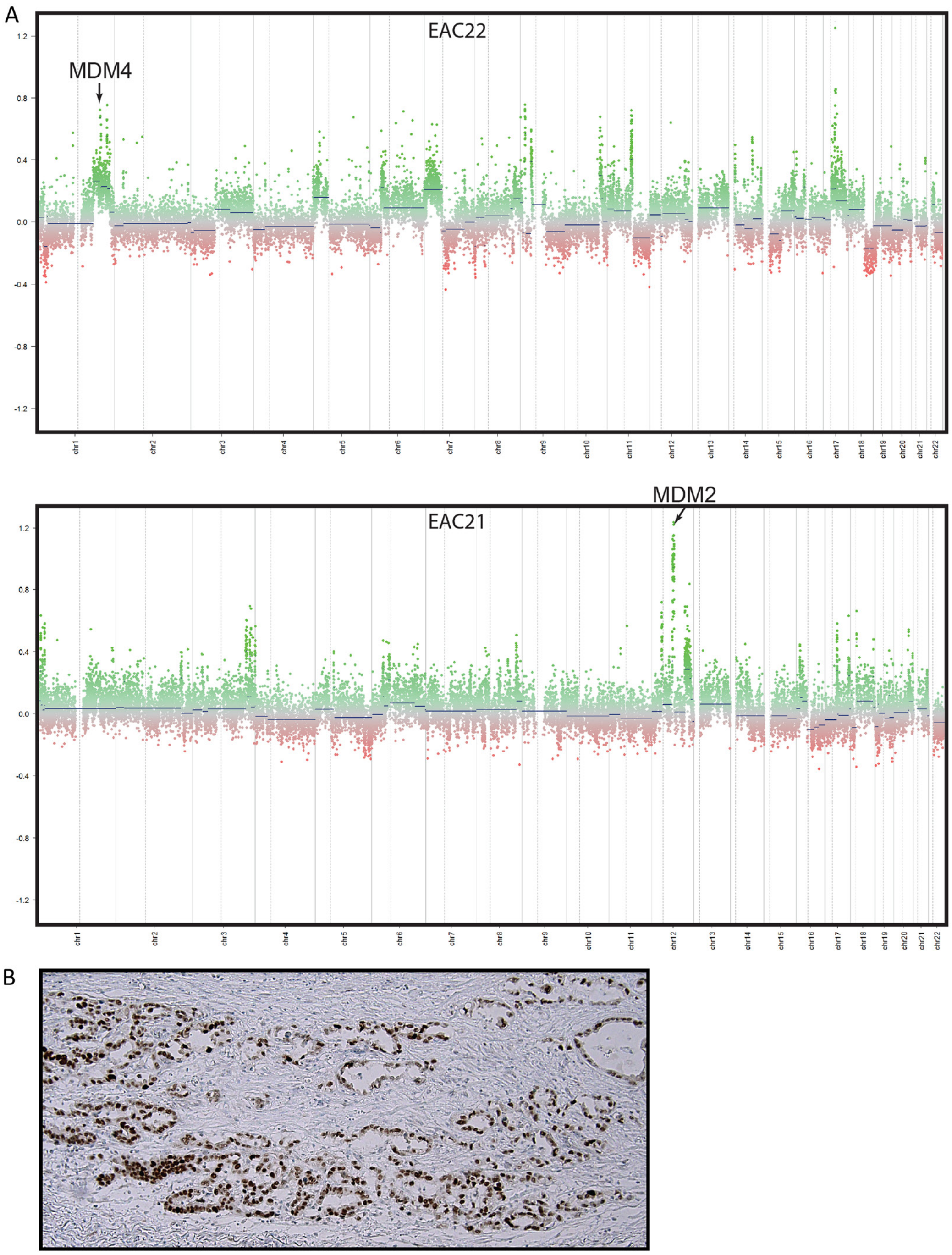

Supplementary Figure 2: (A) Copy number profiles of selected esophageal adenocarcinoma (EAC) cases (sample number 21 and 22). **(B)** MDM2 immunohistochemistry confirming amplification (sample number 21). A: Methylation intensity data were used to calculate relative copy numbers (output of the Conumee software package). Under- (red) and over-represented (green) regions are highlighted. The positions of the *MDM2* and *MDM4* amplicon peaks are indicated. The blue lines represent the regions within chromosomes (segments) with similar copy number. The total number of segments was determined per sample to estimate the relative frequency of intrachromosomal breaks in each case.B: MDM2 amplification of sample number 21 was validated by immunohistochemical staining, magnification 100x.

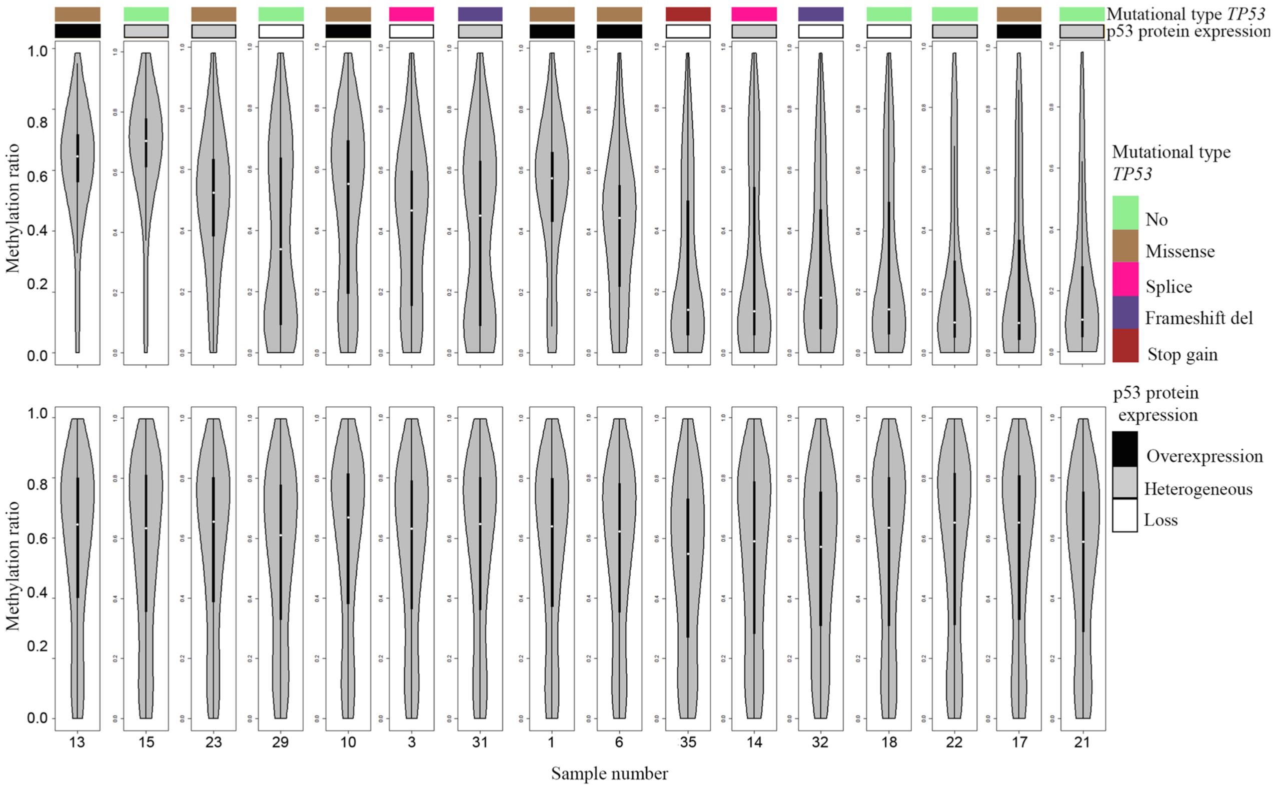

Supplementary Figure 3: Violin plots of the selected and overall CpG methylation data. Upper panel: the methylation profile of the top 10,545 differental probes; lower panel: the methylation profile of all available methylation probes used, indicating no significant differences between cases based on all available methylation data. Abbreviation: Frameshift del: Frameshift deletion.

Supplementary Table 1: Basic clinico-pathological characteristics for all patients subjected to mutational analysis

	TP53 sequencing (n=33)	
	**N**	**%**
Age at surgery		
Median	63.00	
Range (IQR)	54.50-71.50	
Sex		
Male	31	93.9
Female	2	6.1
Siewert classification		
Type 1	12	36.4
Type 2	21	63.6
Pathologic T-stage		
pT2	3	9.1
pT3 or pT4	30	90.9
Pathologic N-stage		
pN0	6	18.2
pN1 or more	27	81.8
Histology grade		
Well	1	3.0
Moderate	10	30.3
Poor	22	66.7
Resection margin status		
pR0	22	66.7
pR1	11	33.3
Follow-up time, months		
Median	19.8	
Range (IQR)	9.63-41.33	
p53 expression		
0%	10	30.3
1-60%	13	39.4
61-100%	10	30.3

Supplementary Table 2: Calculation of optimal cut-off for % nuclear positive tumor cells for p53 immunohistochemistry

% of p53 positive tumor cells	Sensitivity	Specificity	Youden-index
1-20	1	0	0
21-40	0,944	0,127	0,071
41-60	0,894	0,206	0,1
61-80	0,866	0,27	0,136
81-90	0,775	0,317	0,092
91-100	0,69	0,397	0,087
0	0,254	0,698	-0,048

Supplementary Table 3: Univariate Cox regression analysis for disease free survival and overall survival in neoadjuvant treatment naïve patients with esophageal adenocarcinoma

	Univariable Cox regression analysis					
	Disease free survival			Overall survival		
	HR	95% CI	p-value	HR	95% CI	p-value
**Age**	1.007	0.990-1.024	0.412	1.017	1.002-1.033	0.026
**Sex (ref male)**<br>**Female**	1.172	0.750-1.833	0.486	0.942	0.612-1.451	0.786
**Weight**	0.990	0.977-1.003	0.142	0.992	0.981-1.004	0.171
**Siewert**<br>**(ref Type I)**<br>**Type II**	0.859	0.612-1.205	0.379	0.871	0.639-1.186	0.380
**pT-stage**<br>**(ref pT2)**<br>**pT3/4**	2.723	1.503-4.932	0.001	2.394	1.427-4.014	0.001
**pN-stage**<br>**(ref pN0)**<br>**pN+**	3.504	2.044-6.007	<0.001	2.460	1.602-3.778	<0.001
**Differentiation**<br>**(ref well to moderate)**<br>**Poorly**	1.716	1.216-2.421	0.002	1.544	1.134-2.104	0.006
**Resection margin**<br>**(ref pR0)**<br>**pR+**	2.143	1.528-3.005	<0.001	2.101	1.540-2.867	<0.001
**p53**<br>**(ref heterogeneous)**<br>**Loss of expression**	1.569	0.897-2.743	0.036	1.333	0.831-2.138	0.265
**Overexpression**	1.909	1.161-3.139		1.420	0.931-2.165	

HR=Hazard Ratio, CI=Confidence Interval, patients who died within one month of surgery were excluded, p53 immunohistochemistry assessed as loss (0% of the tumor cells positive), heterogeneous expression (1-60% of the tumor cells positive) and overexpression (61-100% of the tumor cells positive).

Supplementary Table 4: Summary of mutations found by Ion Torrent Sequencing of our custom made gene panel.

See Supplementary File 1

Supplementary Table 5: Number of *TP53* mutations and other mutations (*SMAD4*, *ARID1A*, *PIK3CA*, *DOCK2*, *ELMO* and *CDKN2A*) found by Next Generation Sequencing specified by tumors with aberrant (combined loss of expression and overexpression) and heterogeneous expression of p53 immunohistochemistry (IHC). Difference is calculated by Fisher exact test

	p53 IHC expression		
	Loss and overexpression	Heterogeneous	p-value
TP53 mutated	18	7	0.035
TP53 not mutated	2	6	
Other mutations	7	10	0.032
No other mutations	13	3	

Supplementary Table 6: Characteristics of various earlier studies on the prognostic value of p53 in patients with esophageal adenocarcinoma (EAC)

First Author [Reference]	Year	n EAC (% of total included patients)	Specimen	CRT	p53 clone antibody used	Cut-off for over expression	Loss of expression considered?	Predictive
Flejou[29]	1994	62 (100%)	Resection	No	DO7	NA	No	No
Aloia[25]	2001	44 (72%)	Resection	No	PAb1801	NA	No	Yes
Falkenback[27]	2008	59 (100%)	Resection	No	DO7	5%	No	No
Madani[22]	2010	142 (100%)	Resection	No	DO7	1%	No	Yes
Cavazzola[26]	2009	46 (100%)	Resection	No	DO7	10%	No	No
Lehrbach[21]	2009	75 (100%)	Resection	No	DO7	<2 on scale of 5	No	No
Fareed[28]	2010	245 (94%)	TMA	Yes	NA	10%	No	Yes*
Duhaylongsod[50]	1995	42 (100%)	Resection	Yes	PAb1801	NA	No	No
Sauter[37]	1995	24 (100%)	Biopsy +resection	Yes	PAb1801	>5 adjacent cells in 1HPF	No	Yes
Moskaluk[30] §	1996	88 (100%)	Resection	Yes	DO7	50%	No	No
Wu[33] §	1998	92 (100%)	Resection	Yes	DO7	50%	No	No
Ribeiro[40]	1998	42 (74%	Resection	Yes	DO7	Weak positive	No	No

*: in patients treated with chemotherapy p53 was not predictive for survival. §: Overlap in patient group. NA: Not Available. CRT: chemoradiotherapy.
